# Long-term effects of the COVID-19 pandemic for patients with cancer

**DOI:** 10.1007/s11136-024-03726-9

**Published:** 2024-07-03

**Authors:** Yana Debie, Ziyad Palte, Haya Salman, Lise Verbruggen, Greetje Vanhoutte, Siddharth Chhajlani, Silke Raats, Ella Roelant, Timon Vandamme, Marc Peeters, Peter A. van Dam

**Affiliations:** 1 Multidisciplinary Oncological Center Antwerp (MOCA), Antwerp University Hospital, Drie Eikenstraat 655, Edegem, 2650 Belgium; 2 Center for Oncological Research (CORE), Integrated Personalized and Precision Oncology Network (IPPON), University of Antwerp, Universiteitsplein 1, Wilrijk, 2610 Belgium; 3 Faculty of Medicine and Health Sciences, University of Antwerp, Universiteitsplein 1, Wilrijk, 2610 Belgium; 4 Clinical Trial Center (CTC), Antwerp University Hospital, Drie Eikenstraat 655, Edegem, 2650 Belgium

**Keywords:** Long COVID, COVID-19, SARS-CoV-2, Quality of life, Oncology

## Abstract

**Introduction:**

Long COVID is defined as the continuation of symptoms, unexplainable by alternative diagnosis, longer than four weeks after SARS-CoV-2 infection. These symptoms might hinder daily activities and overall well-being, ultimately impacting quality of life (QoL). Several studies have reported fatigue as the most common symptom, followed by dyspnoea, headache and myalgia. Although it is assumed that long COVID affects 10–20% of SARS-CoV-2 infected individuals, recently numbers up to 60% were described for patients with cancer. This study uncovers the impact of the COVID-19 pandemic on QoL of patients with cancer and how long COVID manifests in this cohort.

**Methods:**

A group of 96 patients with cancer was followed from March 2022 till March 2023. Online questionnaires assessing symptoms associated with long COVID, anxiety and depression (HADS), quality of life (EORTC-QLQ-C30) and cognitive functioning (CFQ) were sent every three months during this period. Furthermore, a semi-structured focus group was organised for qualitative data collection.

**Results:**

Overall, these patients reported a negative impact of the enforced COVID-19 restrictions on the emotional and psychological wellbeing. Forty nine patients with cancer (51.0%) were infected with SARS-CoV-2 over the course of the study, of which 39 (79.6%) reported long COVID symptoms. The most commonly reported symptoms were myalgia (46.2%), fatigue (38.5%) and disturbed sleep (35.9%) and it was observed that male sex is associated with poor long COVID outcomes.

**Conclusion:**

While patients with cancer experience similar long COVID symptoms as healthy controls, the prevalence is remarkably higher possibly due to their compromised immune system and weakened physiological reserve.

**Supplementary Information:**

The online version contains supplementary material available at 10.1007/s11136-024-03726-9.

## Abstract (plain language summary)

Since the outbreak in Wuhan (China) at the end of 2019, the Coronavirus Disease 2019 (COVID-19) pandemic has caused instability at various levels of society. While most patients completely recover from their SARS-CoV-2 infection, 10–20% of infected persons and up to 60% of infected patients with cancer develop long COVID. Long COVID is defined as the continuation of symptoms, which cannot be explained by alternative causes, that last longer than four weeks after initial infection. Even though it is generally accepted that patients with cancer are at increased risk of developing severe COVID-19, it is still unclear how long COVID manifests and whether long COVID impacts quality of life in this cohort. Hence, this study observed that patients with cancer reported a negative impact of the enforced COVID-19 restrictions on the emotional and psychological wellbeing. While patients with cancer experience similar long COVID symptoms as healthy controls, the prevalence is remarkably higher possibly due to their compromised immune system and weakened physiological reserve.

**Supplementary Information:**

The online version contains supplementary material available at 10.1007/s11136-024-03726-9.

## Introduction

Since the outbreak in Wuhan (China) at the end of 2019, the Coronavirus Disease 2019 (COVID-19) pandemic has caused instability at various levels of society [1]. To contain this pandemic, entire countries went into lockdown, aiming to fight the spread of severe acute respiratory syndrome coronavirus 2 (SARS-CoV-2). However these large-scale measures also impacted lifestyle, social and work related interactions [2]. Despite all restrictions, more than 771 million cases of COVID-19 have been reported to the World Health Organization [3]. It is generally accepted that patients with cancer are at increased risk of developing severe COVID-19 [4, 5]. Thus, they experienced a range of vulnerabilities arising from the somatic and infection related consequences of SARS-CoV-2 infection. Besides this, the pandemic also impacted mental health and quality of life (QoL) of patients with cancer due to the disruption in healthcare services and (self)-isolation measures [6–10]. Indeed, previous literature has reported that patients with cancer who contract COVID-19 may not only experience worsened physical symptoms, they also suffered from increased psychological distress, and diminished social functioning, all contributing to a reduced QoL [11, 12]. Finally, patients with cancer are at increased risk of developing long COVID. Some studies report that up to 60% of infected patients with cancer develop long COVID [13–15], while this is only 10–20% in the general population [16, 17].

Long COVID is defined as the continuation of symptoms, which cannot be explained by alternative causes, that last longer than four weeks after initial infection [18, 19]. In general, the most commonly reported symptoms are fatigue, followed by dyspnoea, headaches and myalgia [20, 21]. Other symptoms include, but are not limited to insomnia and neurological symptoms (vertigo, loss of taste and smell, and depression) [22, 23]. While fatigue and dyspnoea are also reported as main symptoms of long COVID in patients with cancer, also cognitive impairment is commonly reported in this cohort. Long COVID can severely impact QoL and emotional well-being of individuals, as persistent symptoms might hinder daily activities and contribute to increased stress, anxiety, and depression. This is particularly concerning for patients with cancer, who may already be dealing with substantial physical and emotional challenges.

To improve patient care, the impact of the COVID-19 pandemic and long COVID on patients with cancer needs to be investigated. Therefore, a cohort of patients with cancer was followed up during one year through sequential online questionnaires and subsequent focus group interview. This allowed to map the prevalence of long COVID and its clinical presentation in patients with cancer and to get insight in the impact of the COVID-19 pandemic and long COVID and on the QoL of patients with cancer.

## Materials and methods

### Study design and participants

**Fig. 1:**
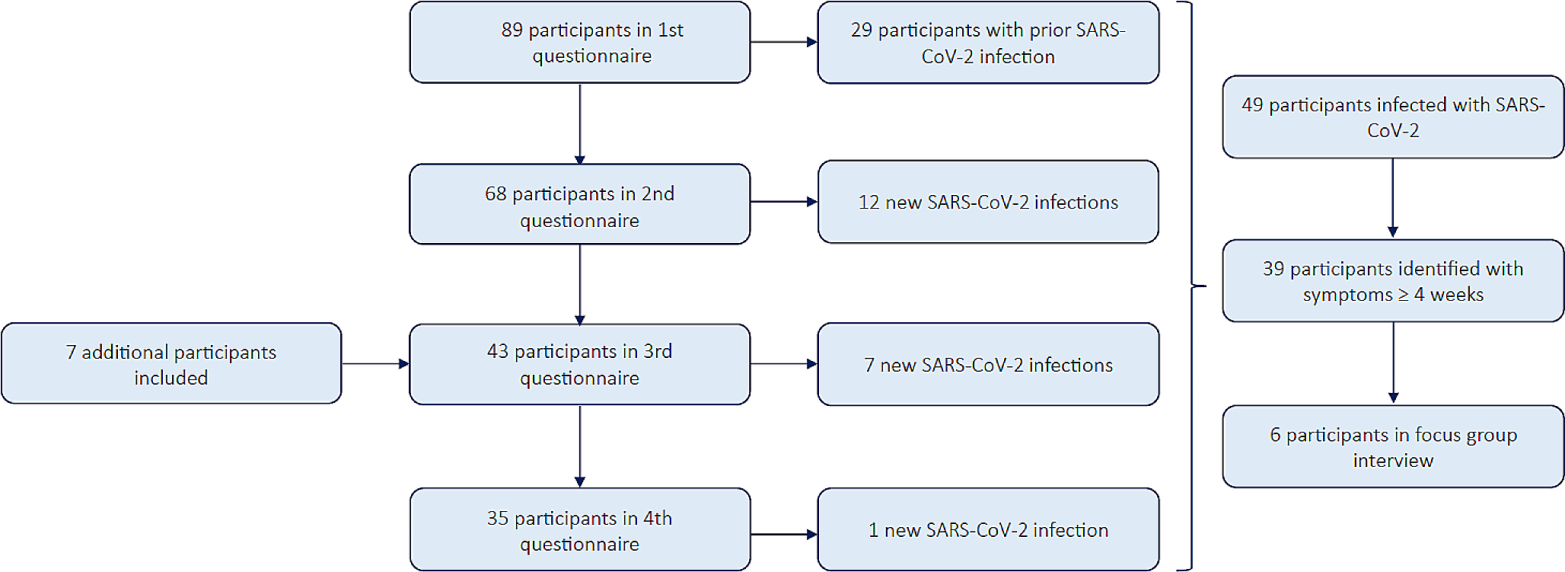
Patient flow diagram

This prospective cohort study was conducted at the Antwerp University Hospital (UZA). All participants were patients with cancer that previously participated in studies centred around humoral immunity in the context of COVID-19, performed at UZA (EudraCT nos. 2021–000300–38, 2021–003573–58 and BUN nr. B3002021000069). Clinical data and data regarding humoral immunity obtained within these studies [5, 24–27] were used within this manuscript to assess correlations with long-term effects of COVID-19. Data about long COVID associated symptoms and QoL were collected from March 2022 till March 2023 through a digital questionnaire and was sent to the study participants at four timepoints over the course of one year, with an interval of approximately three months. For analytical purpose, after each questionnaire, the patients were divided in to two groups (patients with confirmed SARS-CoV-2 infection in the past (COVID+) vs. without (COVID-)). When the third questionnaire was completed (December 2022), a subset of patients reporting long COVID symptoms, was invited for participation in a focus group interview (Fig. 1).

The study was approved by the local ethics committee of UZA (EC number 2021 − 0627) and was executed in accordance with Good Clinical Practice (GCP) and the Declaration of Helsinki [ICH GCP E6(R2)].

### Study procedures

#### Questionnaire

A questionnaire was designed to collect pseudonymized data on four dimensions: QoL, symptoms of anxiety and depression, cognitive functioning and most common symptoms associated with long COVID. Besides the direct effect of potential long COVID symptoms on the patients wellbeing, the questionnaires also retrospectively assessed patient experiences on healthcare and policy throughout the pandemic. Additionally, in the first questionnaire personal information including height, weight, smoking and employment status, cancer diagnosis and cancer treatment was collected. The first part of the questionnaire assessed QoL, based on the ‘*European Organization for Research and Treatment for Cancer Quality of Life Questionnaire (EORTC-QLQ-C30)* [28]*’* including EORTC Functional and Symptom Scores. This questionnaire has become the most widely used questionnaire to evaluate the quality of life of patients with cancer. The second part covering symptoms of anxiety and depression was assessed through the *‘Hospital Anxiety and Depression Scale (HADS)’*. The HADS is often used as a screening tool in primary care and is found to perform well in assessing the symptom severity and cases of anxiety disorders and depression. The part focussing on cognitive functioning was surveyed through the *‘Cognitive Failures Questionnaire (CFQ)* [29]*’*, which is a useful instrument for comparison of the subjective cognitive functioning. For analytical purpose, the scores on the different scales were considered as clinical outcomes. Finally, the last part of the questionnaire was based on the ‘*Derbyshire Post Covid Syndrome Questionnaire* [30]’ and the ‘*Newcastle post-COVID syndrome Follow Up Screening Questionnaire* [31]’. These questionnaires were published in The United Kingdom in the beginning of the pandemic and designed to cover the most common symptoms associated with long COVID. Details on the used questionnaires can be consulted in Suppl. File 1.

After completing each questionnaire, long COVID was identified when a patient developed new symptoms during or after SARS-CoV-2 infection, that cannot be explained by alternative causes and persisted longer than four weeks. This was checked with medical chart review.

#### Focus group interview

Qualitative data on the effect of the COVID-19 pandemic and long COVID on the QoL of the participants was gathered by a semi-structured focus group interview. A group of six patients with cancer with long COVID, participated in the focus group interview (Suppl. Table 1). During the interview, four different themes were identified: experience of SARS-CoV-2 infection and long COVID, impact of the pandemic on QoL, behavioural changes and infection prevention, and experience of healthcare throughout the pandemic. Details of the focus group interview are presented in Suppl. File 2. Colaizzi’s phenomenological method was used to analyze the collected data and Nvivo 1.7.1 was used as coding instrument for the transcribed interview [32].

### Statistical analyses

A linear mixed model (LMM) was used to compare outcomes (number of symptoms, EORTC QoL Score, EORTC Functional and Symptom Scores, HADS Anxiety and Depression Scores, and CFQ Score) between COVID-19 subgroups (COVID + vs. COVID-) over time. In addition, time (in essence nr. of the questionnaire: 1 to 4), COVID-19 group, and the interaction between both were included as fixed effects. Subjects were used as random intercepts. Residuals were assessed for systematic patterns and heteroscedasticity. Independent Samples T-tests or, in case assumptions were not met, Mann-Whitney U tests were performed to compare outcomes between COVID-19 groups. The same tests were performed to compare outcomes between gender and smoking groups within the long COVID group. Risk factors for the development of long COVID were identified with the use of a Fisher Exact test. Spearman correlation was calculated between continuous variables. A two-sided P-value < 0.05 was considered statistically significant. A two-sided P-value between 0.05 and 0.10 was considered to indicate a trend towards significance. Data analysis was done with IBM SPSS v28, SAS version 9.4 and R version 4.1.2.

## Results

### Impact of the COVID-19 pandemic on quality of life

A group of 96 patients was included in the study. The mean age of the participants was 62.6 ± 10.2 years and female participants represent 64.6% of the study population. Detailed demographics are presented in Suppl. Table 2. A LMM analysis was performed to examine whether the outcomes (number of symptoms, EORTC QoL Score, EORTC Functional and Symptom Scores, HADS Anxiety and Depression Scores, and CFQ Score) changed during the one year follow-up and whether the scores differed significantly between the COVID groups at each timepoint (questionnaires 1 through 4) (Table 1). It was observed that nor time or COVID group, nor the interaction between both had a significant impact on any of the outcome measures (Suppl. Tables 3 and 4).

However, in the focus group interview all participants stated that they faced challenges due to COVID-19 restrictions during the pandemic. These restrictions negatively affected their possibility to rely on friends and family for emotional and psychological support, ultimately reducing their emotional wellbeing. The participants also experienced feelings of loneliness and frustration, especially during the different periods of lockdown. These feelings might be the cause of the continued low QoL and mental health of the focus group participants. Some patients noted changes in their relationships with friends and family during the pandemic. Patients perceived themselves as vulnerable with increased risk for complications, resulting in behavioral changes to avoid SARS-CoV-2 infection. Apart from the sense of vulnerability, one participant stated that he feels a certain responsibility to minimize his infection risk. How patients experienced the impact of the pandemic on healthcare differs from one individual to another. Due to COVID-19 restrictions, individual attendance at appointments and treatments, without the presence of loved ones, was required. One participant experienced an improved healthcare efficiency during the pandemic.

**Table 1:** Descriptive statistics of the dependent variables across time

	Survey							
	Survey 1		Survey 2		Survey 3		Survey 4	
	COVID-19		COVID-19		COVID-19		COVID-19	
	Naive	Infected	Naive	Infected	Naive	Infected	Naive	Infected
	Mean ± SD	Mean ± SD	Mean ± SD	Mean ± SD	Mean ± SD	Mean ± SD	Mean ± SD	Mean ± SD
Number reported symptoms	2.7 ± 2.5	2.7 ± 1.7	3.0 ± 2.8	2.5 ± 2.0	2.2 ± 1.8	2.8 ± 2.3	2.5 ± 2.1	1.8 ± 2.4
EORTC QoL Score	72.9 ± 19.1	71.3 ± 18.4	69.8 ± 23.6	71.8 ± 19.1	70.5 ± 21.7	71.9 ± 16.5	67.1 ± 22.8	78.4 ± 18.2
EORTC Functional Score	80.3 ± 19.0	85.4 ± 12.3	78.3 ± 19.7	84.0 ± 11.4	78.4 ± 18.0	83.6 ± 11.2	76.4 ± 18.2	89.0 ± 13.6
EORTC Symptom Score	14.8 ± 13.2	15.2 ± 12.4	18.5 ± 15.9	16.9 ± 9.1	17.5 ± 14.1	15.4 ± 9.2	19.1 ± 13.1	10.3 ± 10.1
HADS Anxiety Score	5.3 ± 4.0	3.4 ± 2.5	5.9 ± 4.0	4.4 ± 3.1	5.1 ± 3.3	5.2 ± 2.5	5.3 ± 3.4	3.6 ± 2.2
HADS Depression Score	3.8 ± 3.8	1.9 ± 2.2	4.4 ± 4.3	3.3 ± 2.9	3.8 ± 3.5	4.6 ± 3.6	4.8 ± 4.3	3.5 ± 4.6
CFQ Score	24.4 ± 16.1	25.4 ± 15.0	25.6 ± 18.7	28.5 ± 14.6	27.9 ± 17.7	27.5 ± 13.3	31.8 ± 16.1	21.6 ± 14.9
N	60	29	37	31	24	19	18	17

### Long COVID symptoms

**Fig. 2:**
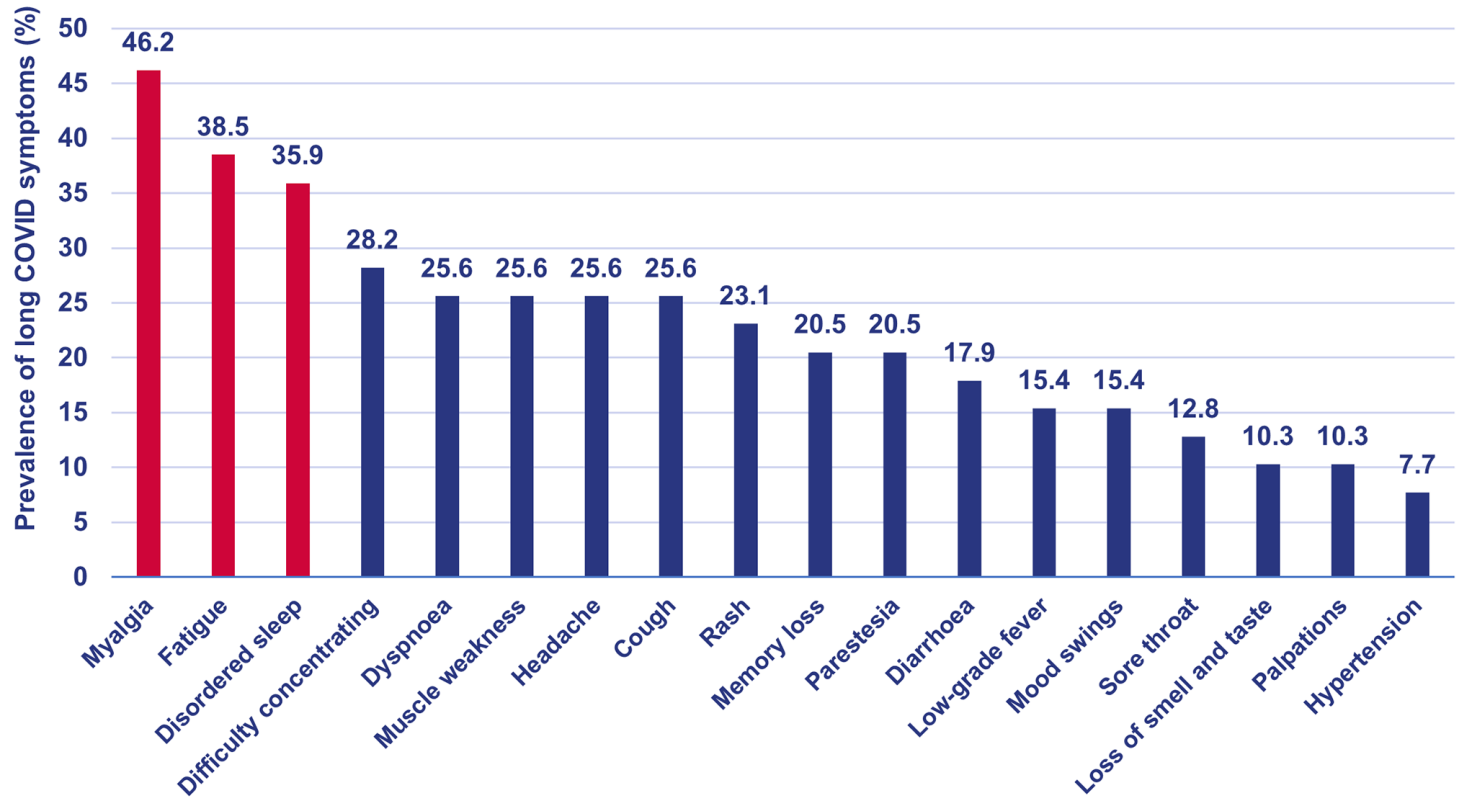
Prevalence of symptoms in patients with cancer with long COVID (*N* = 39). Long COVID symptoms reported by patients with cancer. Percentage per symptom is calculated by the number of patients reporting a specific symptom, divided by total amount of patients with long COVID symptoms (*N* = 39). Red bars indicate a symptom was reported in more than 30% of the patients. Blue bars represent less frequently reported symptoms

Of all patients included in the study, 49 (51%) reported a SARS-CoV-2 infection. Within this COVID + subgroup, long COVID symptoms were registered for 39 (79.6%). In participants identified with long COVID, myalgia (46.2%), fatigue (38.5%) and disrupted sleep (35.9%) were the most commonly reported symptoms (Fig. 2). During the focus group interview patients stated they felt like they did not completely recover after the acute phase of the infection. They noted a change in their health post-infection, but found it challenging to distinguish between long COVID symptoms and the effects of their underlying condition or treatment. Participants also reported that healthcare providers showed minimal interest or did not allocate enough time to address their persisting long COVID symptoms. Although no clear impact of long COVID on the continuity of healthcare was observed by the patients, some patients indicated that they consulted a specialized physician (e.g. pneumologist) in response to their persisting symptoms. This might indicate that long COVID has an impact on healthcare continuity of which patients are not aware. The presence of long COVID might increase the demand for medical services, requiring a multidisciplinary approach and putting pressure on resource allocation.

### Impact of SARS-CoV-2 infection and long COVID on quality of life

Within the COVID + group, LMM subanalyses were performed to compare outcomes between patients who developed long COVID and those that did not. Clinical outcomes and demographics were analysed at the moment long COVID was identified for patients with long COVID and at the first available time point for patients who did not develop long COVID. Significantly worse scores on all assessed scales, with exception for the HADS Anxiety Subscale were reported for long COVID patients (Table 2). Additionally, we observed that a Body Mass Index (BMI) higher than 25.0 increases the risk of developing long COVID after SARS-CoV-2 infection up to 4.5 times (*p* < .1, trend towards significance) indicating that this might be an important risk factor for the development of long COVID in patients with cancer.

**Table 2:** Impact of long COVID on QoL

	Long COVID patients (*N* = 39) Mean ± SD	Infected patients (*N* = 10) Mean ± SD	*p*-value
EORTC QoL Score	68.6 ± 19.45	85.8 ± 12.45	0.011
EORTC Functional Score	83.0 ± 11.70	91.1 ± 12.31	0.019
EORTC Symptom Score	17.0 ± 10.48	6.4 ± 8.65	0.002
HADS Anxiety Score	4.7 ± 3.27	3.3 ± 1.57	0.313
HADS Depression Score	3.5 ± 3.07	0.9 ± 0.88	0.008
CFQ Score	27.9 ± 14.43	13.2 ± 10.04	0.002

Clinical outcomes in SARS-CoV-2 infected patients were compared between patients that developed long COVID (Long COVID patients) and those that didn’t (infected patients) with the use of independent samples T-tests or, in case assumptions were not met, Mann-Whitney U tests. For each group, means and standard deviations (SD) are presented for each outcome

When only looking at the patient group identified with long COVID, subanalyses showed that male patients with long COVID exhibit significant higher HADS Depression Scores compared to females (Mean 5.2 ± 3.25 vs. 2.8 ± 2.77, *p* < .05). Additionally, in patients with long COVID, those who are currently smoking or have a history of smoking report higher HADS Depression Scores than patients that never smoked, that trends towards significance (4.8 ± 3.76 vs. 2.5 ± 2.33, *p* < .1). Patients with comorbidities tend to have more long COVID symptoms compared to patients without comorbidities (4.8 ± 2.09 vs. 3.6 ± 2.15, *p* < .1). Additionally, patients with metastasis have lower QoL compared to patients with non-metastasized cancer (60.7 ± 20.78 vs. 75.6 ± 18.62, *p* < .1). Remarkably, this difference was not observed in the patients groups without long COVID (COVID- and infected without long COVID).

To explore the relationship between continuous variables in long COVID patients, correlation analyses were performed (Table 3). It was observed that patients with more comorbidities, tend to develop more long COVID symptoms (Spearman rank correlation coefficient (r) = 0.296, *p* < .1, trend towards significance) and report higher EORTC Symptom Scores (*r* = .317, *p* < .05), indicating a higher level of symptomatology. Additionally, the number of symptoms is negatively correlated with QoL (*r* = − .347, *p* < .05).

**Table 3:** Correlations between different outcomes in long COVID patients

	Age	BMI	Number of comorbidities	Number of symptoms	EORTC QoL Score	EORTC Functional Score	EORTC Symptom Score	HADS Anxiety Score	HADS Depression Score	CFQ Score
Age										
BMI	0.327**									
Number of comorbidities	0.206	0.258								
Number of symptoms	0.157	0.185	0.296*							
EORTC QoL Score	-0.055	-0.203	-0.243	-0.347**						
EORTC Functional Score	0.052	-0.283*	-0.199	-0.243	0.552**					
EORTC Symptom Score	-0.094	-0.058	0.317**	0.340**	-0.644**	-0.637**				
HADS Anxiety Score	-0.244	0.025	-0.089	-0.014	0.051	-0.236	0.133			
HADS Depression Score	0.022	0.189	0.145	0.181	-0.162	-0.365**	0.142	0.647**		
CFQ Score	-0.201	-0.092	-0.080	0.035	0.024	-0.275*	0.023	0.517**	0.433**	

Spearman correlation between different numeric variables. Level of significance is specified at *p < .1, ** < *p* < .05

**Fig. 3:**
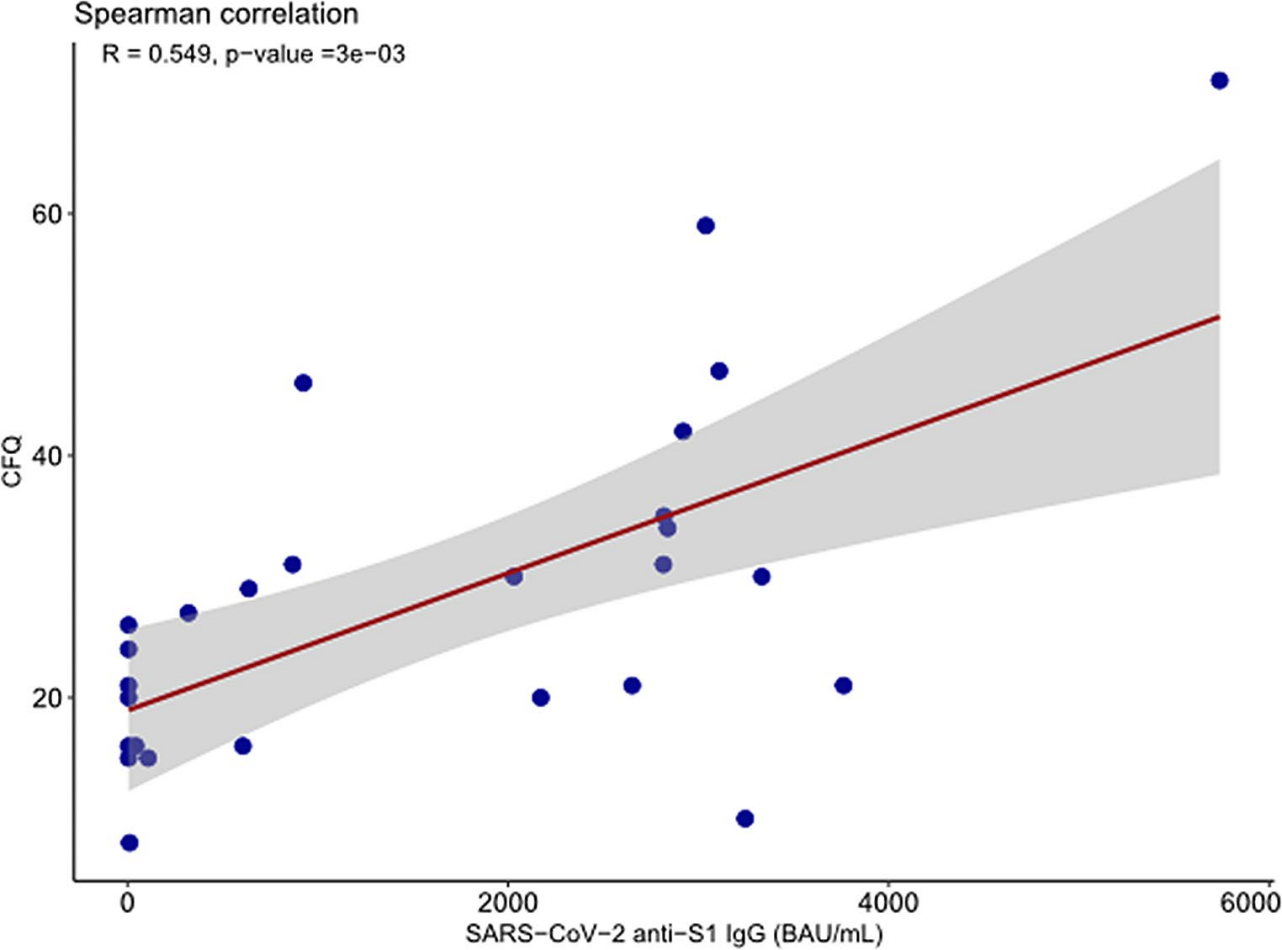
Spearman correlation between anti-S1 IgG antibody titers and CFQ Scores in patients with cancer with long COVID (*N* = 39). Correlation between anti-S1 IgG antibody titers before SARS-CoV-2 infection and CFQ Scores extracted from the first questionnaire after infection. Anti-S1 IgG-class antibody titers were quantified using a SARS-CoV-2 immunoassay, Siemens Healthineers Atellica IM SARS-CoV-2 IgG (sCOVG) assay, for the detection of antibodies (BAU/mL). The measuring interval was 10.90 to 16350.00 BAU/mL. Values below the detection were imputed to half of it (5.45 BAU/mL), values above the measuring interval were imputed to 33% above the upper limit of detection (21,800 BAU/mL)

Possible correlations between anti-S1 IgG SARS-CoV-2 antibody titers and outcomes after SARS-CoV-2 infection were assessed in patients with long COVID (*N* = 39). A moderate correlation between anti-S1 IgG SARS-CoV-2 antibody titers prior to infection and CFQ Scores after infection was observed (*r* = .55, *p* < .05) (Fig. 3). No significant correlations were observed between SARS-CoV-2 anti-S1 IgG antibody titers and the other outcome variables.

## Discussion

We observed that 80% of SARS-CoV-2 infected patients with cancer developed long COVID. While this in line with the observations of other cohort studies that have reported numbers up to 60% in patients with cancer [13, 15, 33], studies in healthy individuals only report numbers up to 30% [34]. Even though the most commonly reported symptoms (myalgia, fatigue, disrupted sleep) are consistent with what is seen in healthy individuals [35, 36], the discrepancy between prevalence of long COVID between patients with cancer and healthy individuals may arise from the fact that patients with cancer may experience side effects from both cancer and cancer treatment which can resemble long COVID. It is estimated that around 40% of patients with cancer experience long-term or delayed effects from the illness or treatment [37]. Indeed, during the focus group interview patients highlighted the challenge to distinguish between long COVID symptoms and the effects of their underlying condition or treatment, making it challenging to assess the impact of COVID-19 symptoms on these patients. Our findings highlight that patients experienced SARS-CoV-2 infection and the COVID-19 pandemic in diverse ways, emphasizing the need for individualized patient care.

In line with the findings of Subramanian and Crook [35, 38], we observed that increased BMI (BMI > 25) might play a substantial role in the development of long COVID. Additionally, our study indicated that long COVID negatively impacts QoL in patients with cancer and that pre-existing comorbidities are associated with decreased QoL in patients with cancer with long COVID. Several studies indicate that female patients with cancer might be more vulnerable for depression and anxiety compared to male [39, 40], but in our study women with long COVID reported lower HADS Depression Scores compared to men. Additionally, we observed that patients with metastatic solid cancer with long COVID expressed lower EORTC QoL Scores compared to patients with non-metastasized cancer. Although, this might be considered normal, this difference was not observed in the non-infected patient group and the group without long COVID development.

During the focus group interview, patients with cancer indicated that SARS-CoV-2 infection had a clinically meaningful impact on their wellbeing and that the imposed restrictions profoundly affected their emotional and psychological states, ultimately affecting their QoL. Patients clearly expressed concerns about the risk of contracting COVID-19 and implemented various behavioural changes to reduce the chances of (re)infection. A cohort study by Ciazynska et al. revealed significantly lower QoL among patients with cancer worldwide during the COVID-19 pandemic, particularly in social, emotional, and cognitive aspects [41]. Furthermore, QoL was uniquely impacted when confronted with public health emergencies, necessitating the attention and support of family and society [41]. Additionally, a recent Danish cohort study revealed that concerns of contracting COVID-19 were associated with lower QoL [42]. Against expectations, the LMM analysis did not indicate a lower QoL among the participants. A larger sample size or longer follow-up period might have revealed significant differences. Another explanation might be that the outcome measures used in the analysis were not sensitive enough to detect changes over time.

The strength of this study is its dual character as it investigates long COVID by including both its symptoms and its effect on QoL in a qualitative and quantitative manner. Other studies have deepened the knowledge regarding long COVID symptoms, whereas our study combined multiple clusters of potential outcomes of long COVID. Additionally, in this study, not only long COVID symptoms, but also anxiety, depression, QoL and cognitive functioning, were assessed. A limitation of our study is the reduction in participant numbers for each subsequent questionnaire, likely due to its extensive and time-consuming nature. Due to this reduced sample size, the power to find significant main or interaction effects in the mixed model will have been significantly lowered and necessitates a cautious interpretation of the results. Furthermore, potential bias from self-reported symptoms needs to be considered. Omicron was the dominant circulating variant during the course of this study. Several studies reported that the Omicron variant caused less severe infection and odds of developing long-term sequela from COVID-19 were lower compared to other variants [43, 44]. Therefore further studies with a longer follow-up time are necessary to compare and identify factors associated with the risk of long-term effects of COVID-19 in patients with cancer. Demographics and the number of vaccinations, which also may contribute to the development of long COVID were not incorporated in the analyses [45, 46]. As several publications have suggested that SARS-CoV-2 vaccines may have a protective effect against the development of long COVID [47, 48], future studies may find it worthwhile to explore this area of research. In addition to this, reinfection may increase the chances of developing persistent COVID-19 symptoms [45]. The presence of different variants, reinfection, as well as the impact of vaccination, have the potential to modify the patients’ condition and should be further investigated. Despite these limitations, our research provides valuable insights into the QoL of patients with cancer during the pandemic.

## Conclusion

In conclusion, this study sheds light on the complex interplay between cancer, COVID-19, and long COVID. While patients with cancer experience similar long COVID symptoms as healthy individuals, the prevalence is remarkably higher. Being overweight might increase the risk for developing long COVID and the presence of long COVID negatively impacted QoL of patients with cancer. This study highlights the need for individualized patient care and suggest that patients with cancer may experience additional challenges due to the COVID-19 pandemic. Long COVID might be more prevalent in patients with cancer because of their compromised immune system and weakened physiological reserve.

## Electronic supplementary material

Below is the link to the electronic supplementary material.

Supplementary Material 1

Supplementary Material 2

## Data Availability

Data are available upon reasonable request by contacting the corresponding author.
