# Supplementary material for: Long-term effects of the COVID-19 pandemic for patients with cancer
Source: Qual Life Res. 2024 Jul 3;33(10):2845–53. doi: 10.1007/s11136-024-03726-9 (PMC11452417; doi:10.1007/s11136-024-03726-9)
Supplement: Supplementary file 1 — Supplementary Material 1 [file 11136_2024_3726_MOESM1_ESM.docx]

Supplementary Material

*Suppl. Table 1: Baseline characteristics of the focus group participants (N = 6)*

| Participant | Sex | Age | Cancer type | Diagnosis (year) | Treatment | Long COVID symptoms |
| --- | --- | --- | --- | --- | --- | --- |
| 1 | F | 61 | Breast | 2020 | Targeted/hormonal therapy | Myalgia, fatigue |
| 2 | F | 66 | Neuro-endocrine tumor | 2020 | Targeted/hormonal therapy | Dyspnea |
| 3 | F | 67 | Lung | 2006 | Immunotherapy | Fatigue, dyspnea |
| 4 | F | 61 | Breast | 2019 | Targeted/hormonal therapy | Fatigue, myalgia, muscle weakness |
| 5 | M | 72 | Hematological malignancy | 2019 | Hematological treatment | Dyspnea |
| 6 | M | 69 | Prostate | 2019 | Targeted/hormonal therapy | Difficulty concentrating, muscle weakness, dyspnea |

***Suppl. Table 2: Patient demographics of the study participants***

*Patients with cancer were assigned to treatment groups based on type of therapy they were receiving at the moment of study inclusion. For patients with hematologic malignancies, a distinction was made between patients receiving B-cell–depleting therapy and all other treatments (stem cell transplantation, targeted therapy, chemotherapy, ed.). Metastasis of hematological malignancies was not assessed and therefore indicated as not applicable. Values are presented as means with standard deviations for continuous variables and as absolute values with percentages for categorical variables.*

|  | **Infected without long COVID development (N=10)** | **Long COVID (N=39)** | **Non-infected (N=47)** | **Overall (N=96)** |
| --- | --- | --- | --- | --- |
| **Gender** |  |  |  |  |
| Female | 6 (60.0%) | 28 (71.8%) | 28 (59.6%) | 62 (64.6%) |
| Male | 4 (40.0%) | 11 (28.2%) | 19 (40.4%) | 34 (35.4%) |
| **Age** |  |  |  |  |
| Mean (SD) | 62.5 (10.5) | 61.6 (9.6) | 63.4 (10.8) | 62.6 (10.2) |
| Median [Min, Max] | 61.5 [47.0, 82.0] | 63.0 [40.0, 79.0] | 64.0 [39.0, 90.0] | 63.0 [39.0, 90.0] |
| **BMI** |  |  |  |  |
| Mean (SD) | 22.5 (2.5) | 26.3 (5.3) | 26.0 (5.8) | 25.8 (5.4) |
| Median [Min, Max] | 22.1 [18.9, 26.8] | 25.3 [19.2, 44.5] | 24.9 [18.3, 43.3] | 24.7 [18.3, 44.5] |
| **Smoking status** |  |  |  |  |
| Current smoker | 0 (0%) | 0 (0%) | 3 (6.4%) | 3 (3.1%) |
| Former smoker | 3 (30.0%) | 13 (33.3%) | 18 (38.3%) | 34 (35.4%) |
| Non-smoker | 6 (60.0%) | 23 (59.0%) | 26 (55.3%) | 55 (57.3%) |
| Unknown | 1 (10.0%) | 3 (7.7%) | 0 (0%) | 4 (4.2%) |
| **Employment** |  |  |  |  |
| Employed | 3 (30.0%) | 13 (33.3%) | 14 (29.8%) | 30 (31.3%) |
| Retired | 5 (50.0%) | 18 (46.2%) | 21 (44.7%) | 44 (45.8%) |
| Unemployable | 2 (20.0%) | 7 (17.9%) | 12 (25.5%) | 21 (21.9%) |
| Unemployed | 0 (0%) | 1 (2.6%) | 0 (0%) | 1 (1.0%) |
| **Type of malignancies** |  |  |  |  |
| Hematological | 4 (40.0%) | 11 (28.2%) | 16 (34.0%) | 31 (32.3%) |
| Solid | 6 (60.0%) | 28 (71.8%) | 31 (66.0%) | 65 (67.7%) |
| **Metastasis** |  |  |  |  |
| Absent | 4 (40.0%) | 14 (35.9%) | 22 (46.8%) | 40 (41.7%) |
| Present | 2 (20.0%) | 14 (35.9%) | 9 (19.1%) | 25 (26.0%) |
| Not applicable | 4 (40.0%) | 11 (28.2%) | 16 (34.0%) | 31 (32.3%) |
| **Therapy** |  |  |  |  |
| B cell depletion | 2 (20.0%) | 2 (5.1%) | 4 (8.5%) | 8 (8.3%) |
| Chemotherapy | 1 (10.0%) | 4 (10.3%) | 8 (17.0%) | 13 (13.5%) |
| Other hematological treatment | 0 (0%) | 6 (15.4%) | 9 (19.1%) | 15 (15.6%) |
| Immunotherapy | 0 (0%) | 3 (7.7%) | 1 (2.1%) | 4 (4.2%) |
| No current treatment | 2 (20.0%) | 5 (12.8%) | 10 (21.3%) | 17 (17.7%) |
| Targeted/Hormonal therapy | 5 (50.0%) | 19 (48.7%) | 15 (31.9%) | 39 (40.6%) |
| **Comorbidities** |  |  |  |  |
| Cardiovascular disease | 1 (10.0%) | 2 (5.1%) | 7 (14.9%) | 10 (10.4%) |
| Hypertension | 0 (0%) | 1 (2.6%) | 4 (8.5%) | 5 (5.2%) |
| Lung disease | 0 (0%) | 3 (7.7%) | 1 (2.1%) | 4 (4.2%) |
| Diabetes | 0 (0%) | 3 (7.7%) | 4 (8.5%) | 7 (7.3%) |
| Auto immune disease | 0 (0%) | 2 (5.1%) | 2 (4.3%) | 4 (4.2%) |
| Other | 1 (10.0%) | 7 (17.9%) | 7 (14.9%) | 15 (15.6%) |

***Suppl. Table 3: The F- and P-values for the linear mixed models***

|  | Model with interaction between COVID-19 status and Surveys 1-4 | | | | Model without interaction between COVID-19 status and Surveys 1-4 | | | | |
| --- | --- | --- | --- | --- | --- | --- | --- | --- | --- |
|  | Interaction between COVID-19 status and Surveys 1-4 | | Effect of survey for COVID-positive group | | | Effect of COVID-19 status | | Effect of survey | |
|  | F | Sig. | F | Sig. | | F | Sig. | F | Sig. |
| Symptoms | 0.540 | 0.656 | 0.93 | 0.426 | | 0.038 | 0.846 | 0.421 | 0.738 |
| EORTC QoL Score | 0.821 | 0.484 | 0.75 | 0.524 | | 0.049 | 0.825 | 0.144 | 0.933 |
| EORTC Functional Score | 0.214 | 0.887 | 0.69 | 0.561 | | 1.908 | 0.169 | 0.680 | 0.566 |
| EORTC Symptom Score | 0.940 | 0.423 | 1.43 | 0.235 | | 0.843 | 0.360 | 1.996 | 0.117 |
| HADS Anxiety Score | 1.768 | 0.156 | 1.33 | 0.266 | | 0.192 | 0.662 | 1.233 | 0.300 |
| HADS Depression Score | 0.677 | 0.568 | 1.67 | 0.176 | | 0.513 | 0.475 | 1.970 | 0.121 |
| CFQ Score | 0.538 | 0.657 | 0.74 | 0.528 | | 1.506 | 0.221 | 1.002 | 0.394 |

***Suppl. Table 4: Coefficients for the linear mixed model***

|  | Symptoms | EORTC QoL Score | EORTC Functional Score | EORTC Symptom Scale | HADS Anxiety Score | HADS Depression Score | CFQ Score |
| --- | --- | --- | --- | --- | --- | --- | --- |
| Intercept | 2.59 | 73.53 | 81.23 | 14.89 | 4.94 | 3.30 | 24.12 |
| COVID-status | 0.21 | -2.30 | 3.62 | -0.69 | -0.86 | -0.60 | 0.91 |
| Survey 2 | -0.01 | -0.96 | -0.08 | 2.98 | 0.36 | 0.61 | -0.67 |
| Survey 3 | -0.13 | -0.84 | -2.15 | 3.20 | -0.45 | 0.40 | 1.48 |
| Survey 4 | -0.04 | -2.94 | -2.16 | 3.85 | -0.87 | 0.84 | 0.42 |
| COVID-status*Survey 2 | -0.17 | 1.05 | -1.08 | -0.14 | 0.33 | -0.05 | 3.18 |
| COVID-status*Survey 3 | 0.09 | 0.56 | -2.07 | -0.72 | 1.72 | 1.07 | 1.67 |
| COVID-status*Survey 4 | -0.77 | 8.42 | 0.82 | -5.43 | 1.43 | 0.30 | 0.74 |

***Suppl. file 1: Used questionnaires***

European Organization for Research and Treatment for Cancer Quality of Life Questionnaire (EORTC-QLQ-C30) including EORTC Functional and Symptom Scores

The EORTC quality of life questionnaire (QLQ) is an integrated system for assessing the health related quality of life (QoL) of cancer patients participating in international clinical trials. The core questionnaire, the QLQ-C30, is the product of more than a decade of collaborative research. Following its general release in 1993, the QLQ-C30 has been used in a wide range of cancer clinical trials, by a large number of research groups; it has additionally been used in various other, non-trial studies.

The content areas covered by the questionnaire reflect the multi-dimensionality of the QoL construct:

- Five functional scales (physical, role, cognitive, emotional, and social)
- Three symptom scales (fatigue, pain, and nausea and vomiting)
- A global health status / QoL scale
- A number of single items assessing additional symptoms commonly reported by cancer patients (dyspnoea, loss of appetite, insomnia, constipation and diarrhoea)
- Perceived financial impact of the disease

Hospital Anxiety and Depression Scale (HADS)

The HADS Scale is commonly used to determine the levels of anxiety and depression that a person is experiencing. It measures core symptoms of anxiety and depression without including physical symptoms. The scale addresses feelings in the past four weeks and consists of an anxiety scale and a depression scale with both 7 items. The higher a patient scores on this questionnaire, the more symptoms they experience.

Cognitive Failures Questionnaire (CFQ)

The CFQ is a questionnaire consisting of 25 items for measuring subjective cognitive functioning, which examines the frequency of everyday cognitive errors. The CFQ is a measure of self-reported failures in perception, memory, and motor function. Responses to all questions tend to be positively correlated, and the whole questionnaire correlates with other recent measures of self-reported deficit in memory, absent- mindedness, or slips of action. A high CFQ score indicates more cognitive failure.

Derbyshire Post Covid Syndrome Questionnaire

The questionnaire was published in The United Kingdom in December 2020 and was initially designed for people in Derbyshire that have had a PCR-confirmed COVID-19 diagnosis or have experienced COVID-19 symptoms (increased temperature, persistent cough, loss of taste and smell,…). The aim of the questionnaire is to find out whether individuals are experiencing persisting COVID-19 symptoms.

Newcastle post-COVID syndrome Follow Up Screening Questionnaire

This questionnaire is designed to screen for the issues that might prompt concern if still present 10- 12 weeks after initial SARS-CoV-2 infection. The questionnaire was published in The United Kingdom in the beginning of the pandemic and designed to cover the most common symptoms associated with long COVID.

***Suppl. File 2: Detailed information of the focus group interview***

**Experience of SARS-CoV-2 and long COVID**

Five out of six patients (83.3%) experienced a mild SARS-CoV-2 infection that presented with flu-like symptoms that lasted approximately one week. However, one patient reported a more severe infection.

*“I was very ill for a couple of weeks and felt very fatigued. I had to stay home and let the disease run its course.” (participant 3)*

After the acute phase of the infection, all patients felt like they did not completely recover.

*“I still experience shortness of breath and notice that I have less energy than before the infection.” (participant 5)*

Other participants reported persistent fatigue, shortness of breath, and difficulty concentrating. They noted a change in their health post-infection, but found it challenging to distinguish between long COVID-symptoms and the effects of their underlying condition or treatment.

### Impact of the pandemic on quality of life

All participants faced challenges due to COVID-19 restrictions during the pandemic, affecting their reliance on friends and family for emotional and psychological support.

*“I was diagnosed with cancer right after the lockdown started. I wanted to tell my family in person about my diagnosis, but instead I had to share the news over the phone. This was very difficult for me.” (participant 2)*

*“Right after my diagnosis, the COVID-19 pandemic forced me to choose between my son and daughter. The restrictions did not allow me to see both in person.” (participant 5)*

The participants also experienced feelings of loneliness and frustration.

*“The social restrictions imposed by the government, were the most difficult part of the pandemic.” (participant 1)*

*“I experienced the pandemic as a very lonely period, which definitely reduced my quality of life.” (participant 3)*

Some patients noted changes in their relationships with friends and family during the pandemic.

*“I lost some friends throughout the pandemic, because it was so difficult to stay in touch with them.” (participant 5)*

*“Due to circulating conspiracy theories, some family members refused vaccination. This caused my relationship with them to change.” (participant 6)*

### Behavioral changes and infection prevention

Patients often implemented preventive measures to avoid SARS-CoV-2 infection. They perceived themselves as vulnerable with increased risk for complications, resulting in behavioral changes.

*‘If I can avoid public transportation, I will. It is too crowded.” (participant 4)*

*“Whenever I use public transportation, I always wear an FFP2 mask and keep distance from other people. I am definitely more cautious than before the pandemic. I think more about things that be used to be taken for granted.” (participant 5)*

Apart from the sense of vulnerability, one participant stated that he feels a certain responsibility to minimize his infection risk.

*“My doctors are doing their very best to keep me alive and control my illness with an expensive treatment. Therefore, I feel responsibility to stay as healthy as I can.” (participant 5)*

### Experience of healthcare throughout the pandemic

How patients experienced the impact of the pandemic on healthcare differs from one individual to another. Due to COVID-19 restrictions, individual attendance at appointments and treatments, without the presence of loved ones, was required.

*“My husband was not allowed to accompany me during my chemotherapy. As a result, I felt I had to fight my disease all alone.” (participant 1)*

One participant experienced an improved healthcare efficiency during the pandemic.

*“The pandemic improved my experience at the hospital. There was more structure and efficiency. Waiting times were reduced and the waiting rooms were never crowded. Before the pandemic, I often had to wait for more than an hour.” (participant 5)*

Participants reported that healthcare providers showed minimal interest or did not allocate enough time to address their persisting long COVID symptoms

*“Last week, I visited my doctor and mentioned that my fatigue after my SARS-CoV-2 infection is not improving. However, he did not address my remarks.” (participant 3)*

*“I feel like the doctors don’t have time for this kind of problem.” (participant 2)*

A number of participants stated the importance of their general practitioner during the pandemic.

*“My general practitioner made a huge difference for me during the pandemic. She followed up on everything and set things in motion when needed.” (participant 4)*

Also, the pandemic motivated one participant to approach her general practitioner to discuss her DNR status.

*“I went to my* general practitioner *to make sure that I would not be ventilated if I developed a severe case of COVID-19.” (participant 3)*
